# Supplementary figures and images for: Characterization of the Flagellar Collar Reveals Structural Plasticity Essential for Spirochete Motility
Source: mBio. 2021 Nov 23;12(6):e02494-21. doi: 10.1128/mBio.02494-21 (PMC8609358; doi:10.1128/mBio.02494-21)

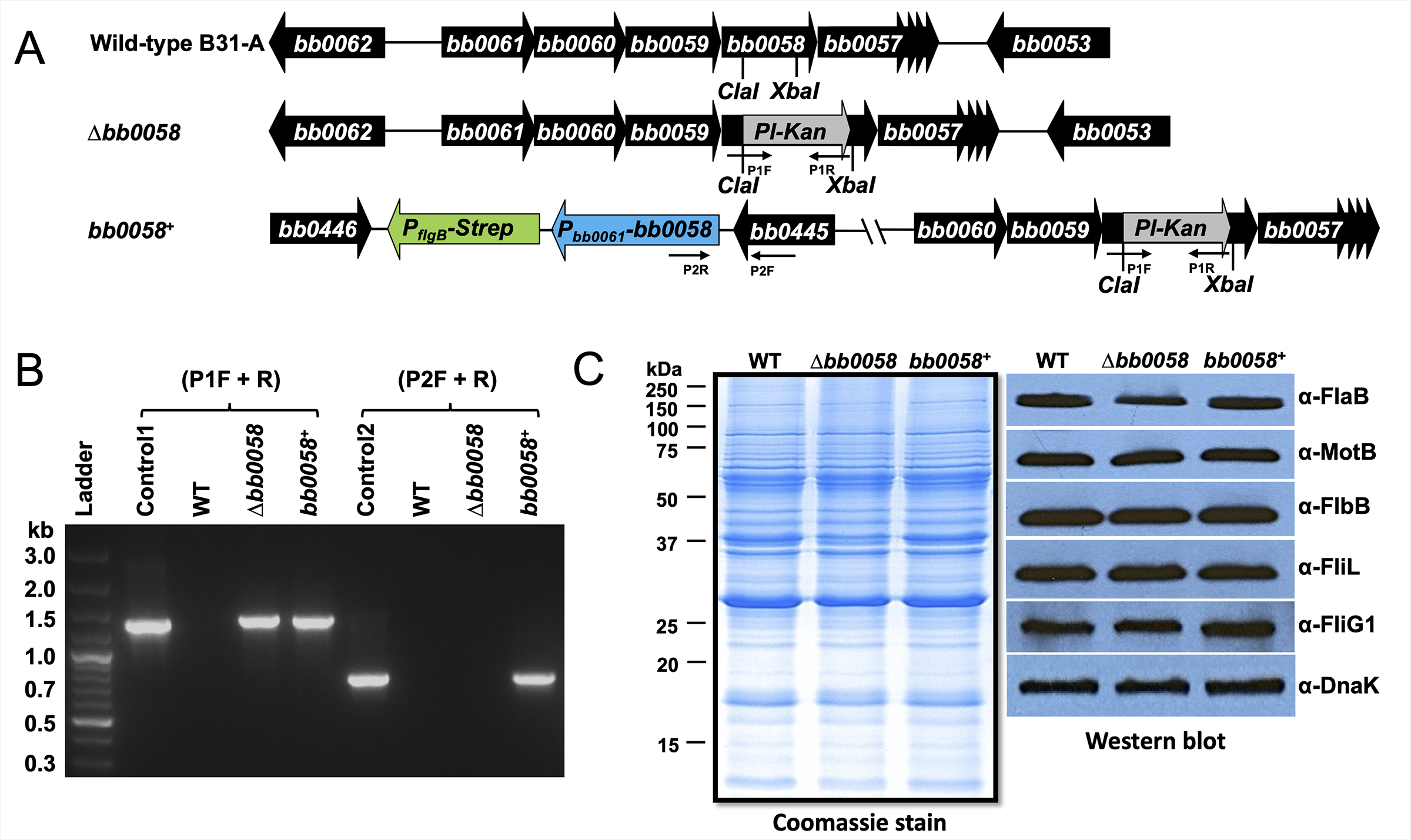

Supplement: FIG S1 [file mbio.02494-21-sf001.tif]

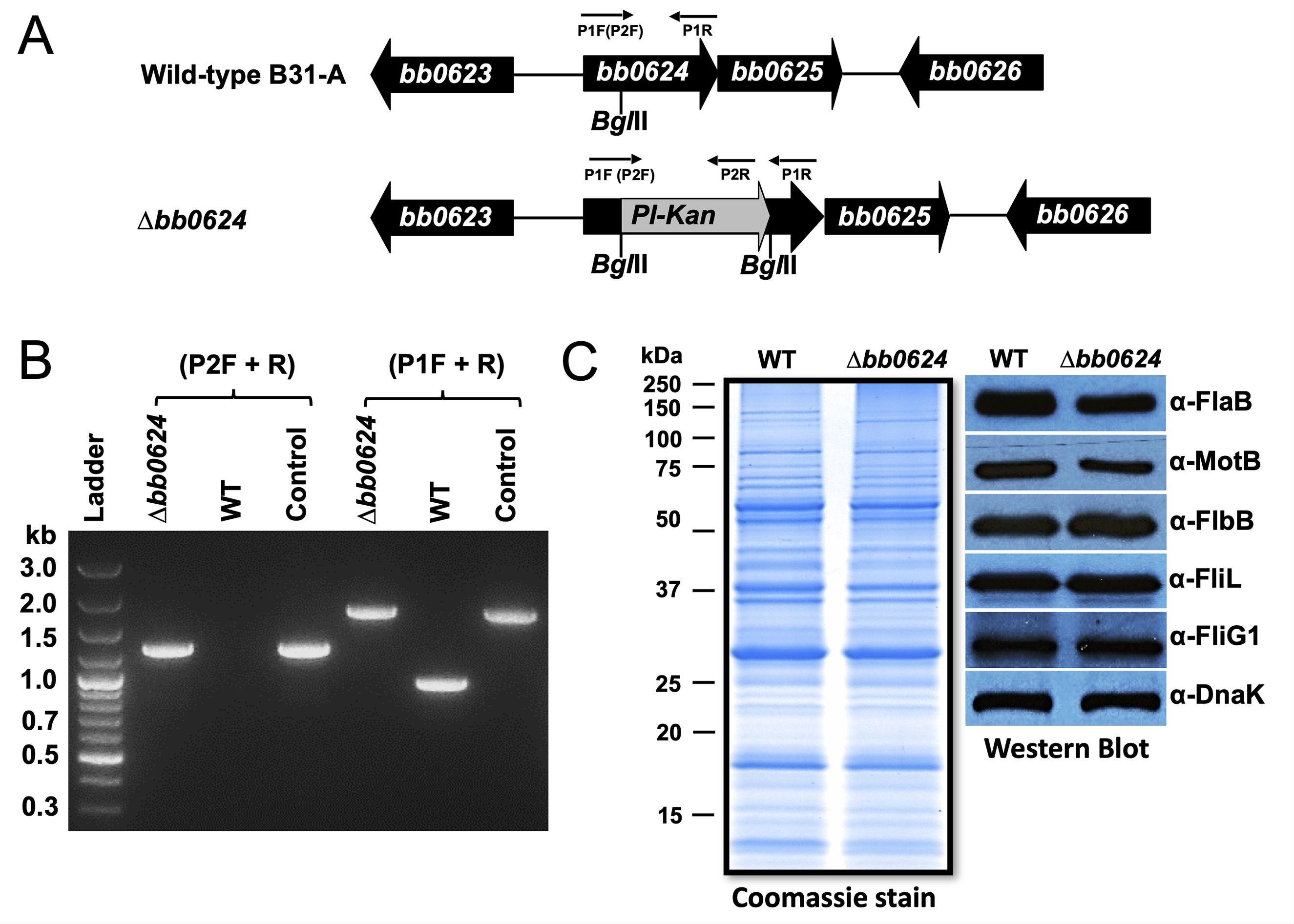

Supplement: FIG S2 [file mbio.02494-21-sf002.tif]

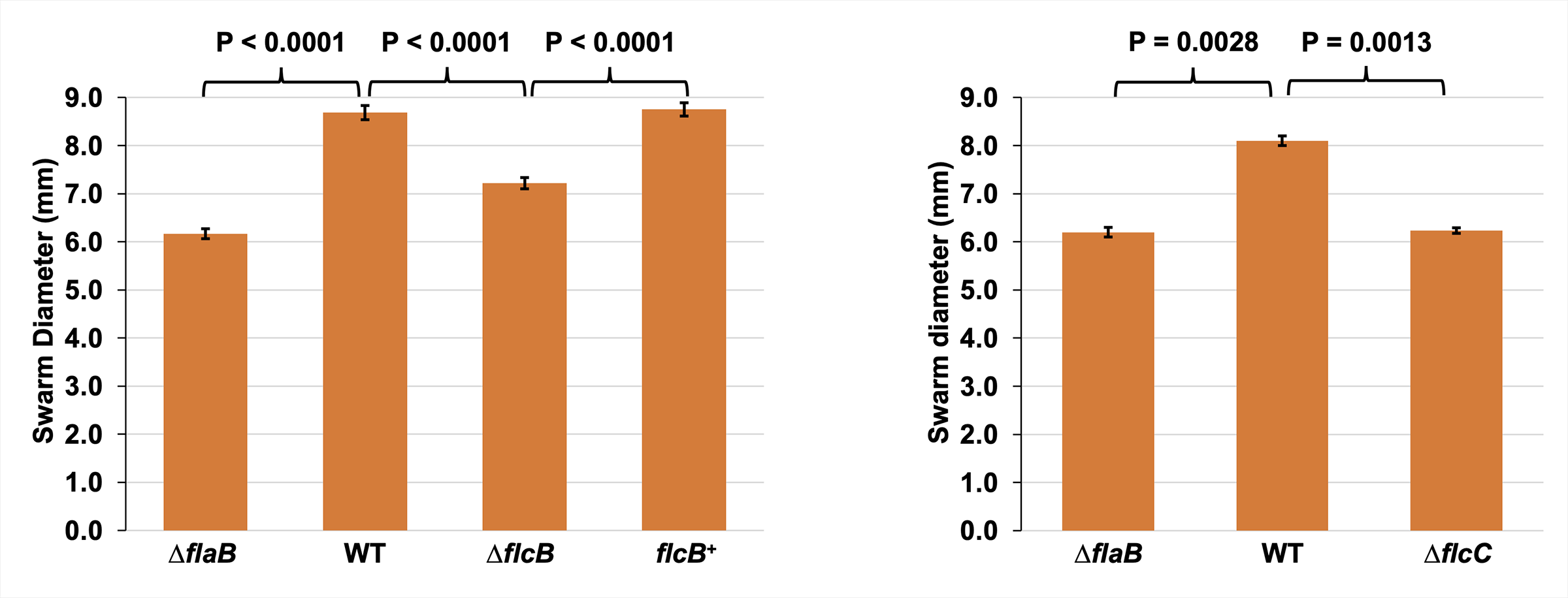

Supplement: FIG S3 [file mbio.02494-21-sf003.tif]

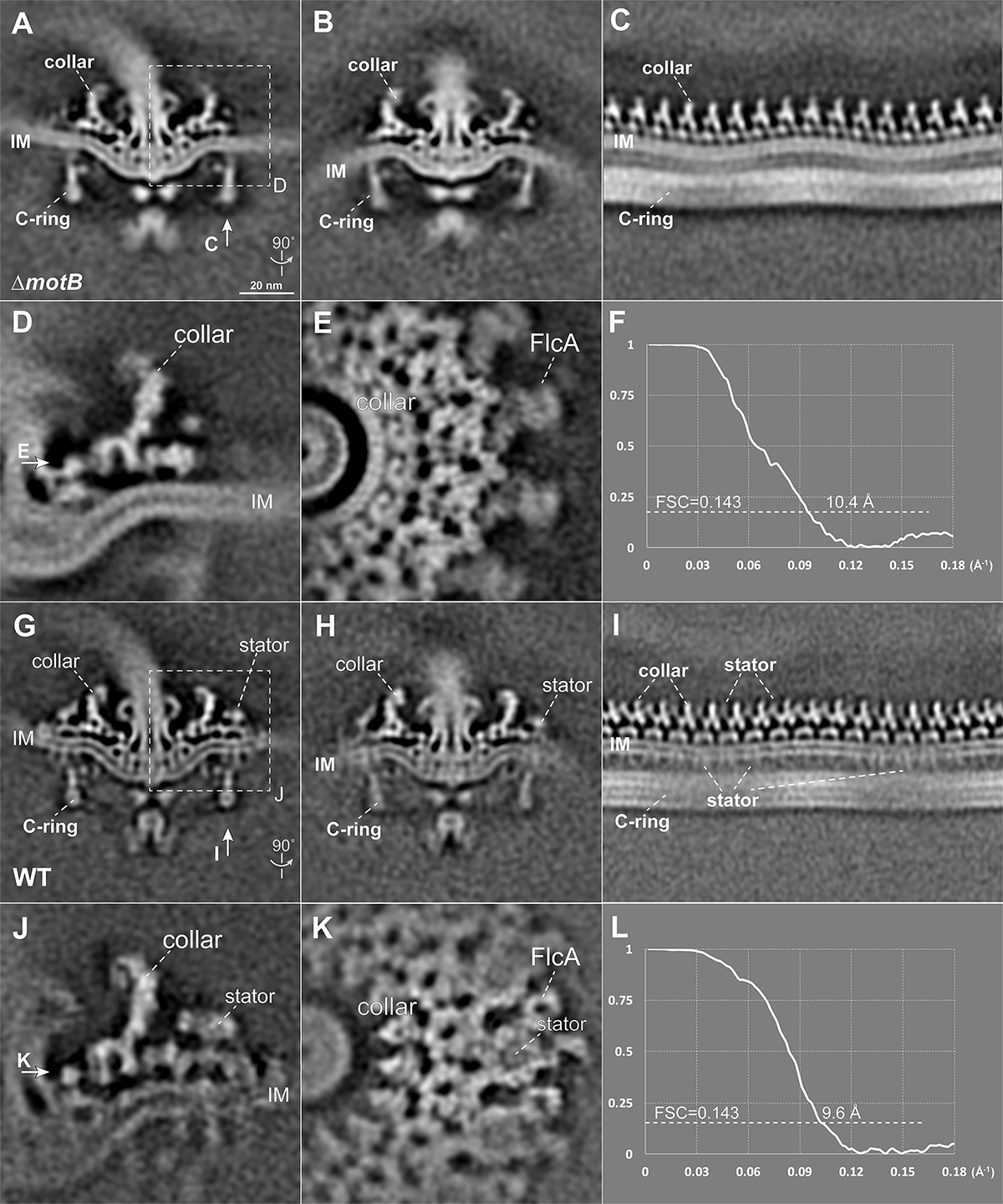

Supplement: FIG S4 [file mbio.02494-21-sf004.tif]

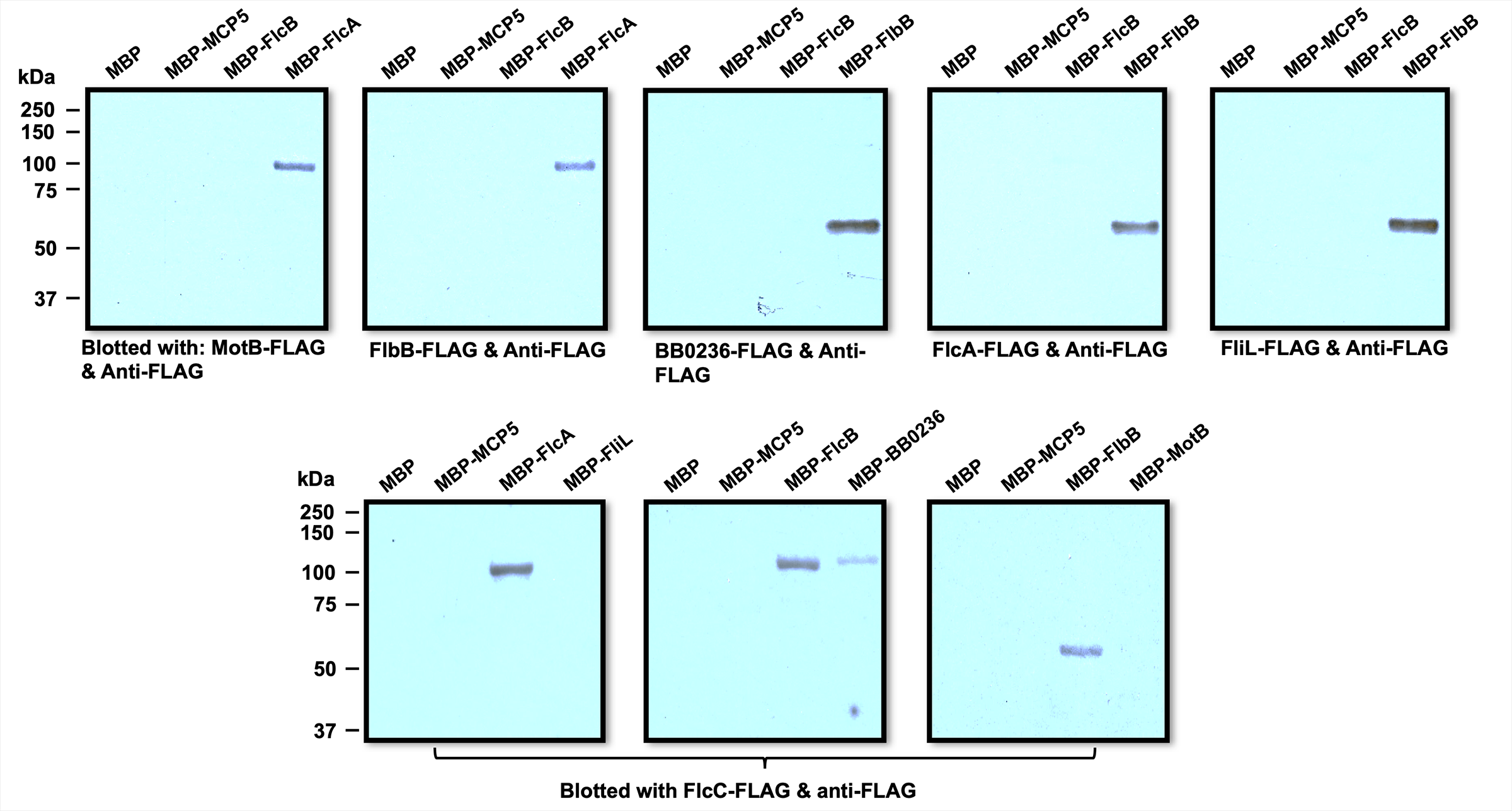

Supplement: FIG S5 [file mbio.02494-21-sf005.tif]
