# Supplementary material for: Characterization of the Flagellar Collar Reveals Structural Plasticity Essential for Spirochete Motility
Source: mBio. 2021 Nov 23;12(6):e02494-21. doi: 10.1128/mBio.02494-21 (PMC8609358; doi:10.1128/mBio.02494-21)
Supplement: TABLE S1 [file mbio.02494-21-st001.pdf]

strain	Camera	Pixel size (Å)	VPP	tilt range	No. of tomograms	No. of motors	No. of collar subunits used for focus refinement
**WT**	K2	2.747	Yes	-51° to 51°	256	1748	5232
			No		189	1202	11928
**Δ*flcB* (Δ*bb0058*)**	K2	3.483	Yes	-51° to 51°	41	172	— —
	K3	3.384		-48° to 48°	132	885	— —
**(*flcB*+) (*bb0058*+)**	K2	1.386	No	-51° to 51°	79	290	— —
**Δ*flcC* (Δ*bb0624*)**	K2	3.483	Yes	-51° to 51°	40	346	5536
	K3	3.384		-48° to 48°	129	798	12256
**Δ*motB***	K2	2.747	Yes	-51° to 51°	233	1448	9427
